# Anti-Inflammatory Effects of *Morinda citrifolia* Extract against Lipopolysaccharide-Induced Inflammation in RAW264 Cells

**DOI:** 10.3390/medicines8080043

**Published:** 2021-08-04

**Authors:** Takashi Tanikawa, Masashi Kitamura, Yasuhiro Hayashi, Natsumi Tomida, Akemi Uwaya, Fumiyuki Isami, Yutaka Inoue

**Affiliations:** 1 Laboratory of Nutri-Pharmacotherapeutics Management, School of Pharmacy, Faculty of Pharmacy and Pharmaceutical Sciences, Josai University, 1-1, Keyakidai, Sakado, Saitama 350-0295, Japan; yinoue@josai.ac.jp; 2 Laboratory of Pharmacognocy, School of Pharmacy, Faculty of Pharmacy and Pharmaceutical Sciences, Josai University, 1-1, Keyakidai, Sakado, Saitama 350-0295, Japan; kitamura@josai.ac.jp; 3 Laboratory of Biological Chemistry, Faculty of Pharma-Science, Teikyo University, 2-11-1 Kaga, Itabashi-ku, Tokyo 173-8605, Japan; hayashiy@pharm.teikyo-u.ac.jp; 4 Research and Development, Morinda Worldwide, Inc., 3-2-2 Nishishinjuku, Shinjuku-ku, Tokyo 160-0023, Japan; Natsumi_Tomida@jp.newage.com (N.T.); Akemi_Uwaya@jp.newage.com (A.U.); Fumiyuki_Isami@jp.newage.com (F.I.)

**Keywords:** morinda citrifolia, noni seeds, NO production, anti-inflammation, RAW264 cells

## Abstract

Leaves of *Morinda citrifolia* (noni) have been used in Polynesian folk medicine for the treatment of pain and inflammation, and their juice is very popular worldwide as a functional food supplement. This study aimed to demonstrate that *M. citrifolia* seed extract exerts anti-inflammatory effects on RAW264 cells stimulated by lipopolysaccharide. To confirm the inhibitory effect of *M. citrifolia* seed extract, we assessed the production of nitric oxide (NO) and inflammatory cytokines. The *M. citrifolia* seed extract showed a significant inhibition of NO production, with no effect on cell viability, and was more active than *M. citrifolia* seed oil, leaf extract, and fruit extract. The *M. citrifolia* seed extract was found to reduce the expression of inducible NO synthase and tumor necrosis factor-alpha of pro-inflammatory cytokines. These results suggest that the anti-inflammatory effect of *M. citrifolia* seed extract is related to a reduction in the expression of inflammatory mediators and support its potential therapeutic use.

## 1. Introduction

Inflammation plays an important role in the progression of numerous pathologies, including cardiovascular disease, cancer, and autoimmune diseases [1,2,3]. The inhibition of inflammatory responses has become a part of the therapy for these diseases.

Macrophages are important in the pathological process of inflammation and have three primary functions in inflammatory responses: antigen presentation, phagocytosis, and the production of various cytokines and active substances that regulate immune functions, including tumor necrosis factor-alpha (TNF-α), interleukin-1 beta (IL-1β), and nitric oxide (NO) [4,5,6]. Inhibiting the production of proinflammatory mediators by activated macrophages might aid the prevention or treatment of inflammation. Several chemical medicines are used for the treatment of inflammatory diseases; however, their use is limited due to the potential for severe side effects. Thus, natural products derived from edible plants with anti-inflammatory activity are being studied. Extracts derived from these plants are considered to have immunomodulatory effects [7].

*Morinda citrifolia* L. (Rubiaceae), commonly known as noni, is a small tropical tree that grows throughout Polynesia. It has been used in folk medicine for more than 2000 years [8]. The leaves, fruit, bark, and roots of the plant have several pharmacological properties [9]. Noni has antibacterial [10], anticancer [11], and anti-inflammatory properties [12]. In 2002, noni juice was accepted in the European Union as a novel food, and the noni juice market has continued to expand [12]. Noni leaves have various benefits [13]. Noni fruit contain a large number of seeds [14]. However, during the production of noni fruit juice, the seeds are removed and discarded. A previous report revealed that noni leaf extracts inhibited inflammatory mediator secretion [15]. However, the effect of *M. citrifolia* seed extract (MCS-ext) during inflammatory reactions is unknown. Therefore, we investigated the utility of the noni seeds.

The aim of this study was to investigate the therapeutic potential of MCS-ext against inflammation. We used RAW264 cells as an in vitro inflammatory model. RAW264 cells are a murine macrophage cell line [16], and lipopolysaccharide (LPS)-stimulated RAW264 cells have been frequently used to investigate macrophage activation in inflammatory diseases. We studied the effects of the extract on the production of TNF-α and NO in LPS-induced RAW264 macrophages.

## 2. Materials and Methods

### 2.1. Plant Materials

Fruit and leaf juice of *M. citrifolia* were obtained from Morinda Inc. (American Fork, UT, USA). *M. citrifolia* leaves were collected from the Society Islands of French Polynesia. These were identified by local Polynesians and confirmed by a native botanist. A voucher specimen (sample code AKP207) was deposited at the Tahitian Noni International Research Center, American Fork, UT, USA. Then, 5 kg of fresh leaves were pressed in a cloth copra press to extract 2.25 kg *M. citrifolia* leaf juice, which was frozen for later use [17]. *M. citrifolia* fruit were harvested in French Polynesia and allowed to ripen fully. The fruit was then processed into a puree by mechanical removal of the seeds and skin via a micro-mesh screen in a commercial fruit pulper, followed by pasteurization (87 °C for 3 s) at a good manufacturing certified fruit processing facility in Mataiea, Tahiti. The pasteurized puree was filled into aseptic containers and stored under refrigeration. This puree was used to concentrate *M. citrifolia* juice. This was done by separating the noni fruit pulp from the juice by centrifugation. The clarified juice was concentrated by evaporation under vacuum [18], and the *M. citrifolia* fruit and leaf juices were freeze-dried. *M. citrifolia* seed extract and oil were obtained from Oryza Oil & Fat Chemical Co., Ltd. (Aichi, Japan). All the samples were dissolved in dimethyl sulfoxide (DMSO) and diluted with the experimental medium. The final concentration of DMSO was 0.1% *v*/*v*.

### 2.2. Reagents

Isogen II was purchased from Nippon Gene Co., Ltd. (Tokyo, Japan). The FastGene Scriptase II cDNA Synthesis kit was purchased from NIPPON Genetics Co., Ltd. (Tokyo, Japan). DMSO, Dulbecco’s modified Eagle’s medium (DMEM; high glucose) without L-glutamine and phenol red, L-glutamine, LPS from *E. coli* O111, MEM nonessential amino acids (NEAA), Eagle’s minimum essential medium (EMEM) with L-glutamine and phenol red, N-1-Naphthylethylenediamine dihydrochloride, penicillin-streptomycin solution, phosphoric acid, and sulfanilamide were purchased from FUJIFILM Wako Pure Chemical Corporation (Osaka, Japan). Fetal bovine serum (FBS) was purchased from Biowest (Nuaille, France).

### 2.3. Cell Culture System

The mouse macrophage-like cell line RAW264 was provided by the RIKEN BRC. RAW264 cells were grown at 37 °C in EMEM containing 10% FBS, 1% NEAA, 100 U/mL of penicillin, and 100 μg/mL streptomycin. For the experiment, RAW264 cells were plated at a density of 5 × 10^4^ cells/well in 96-well plates or 5 × 10^5^ cells/well in 24-well plates in DMEM (without phenol red) supplemented with 0.5% FBS, 2 mM L-glutamine, 100 U/mL of penicillin, and 100 μg/mL of streptomycin. The cells were cultured overnight at 37 °C in a humidified atmosphere of 5% CO_2_/95% air and then treated with test samples for 0.5 h before stimulation with LPS. After culturing with the test samples and LPS, the cell culture supernatant was collected by centrifugation. The concentration of NO in the cell culture supernatant was determined using the Griess reagent system. The cell culture supernatant was mixed with equal volumes of Griess reagent (2.5% *v*/*v* phosphoric acid containing 1% *w*/*v* sulfanilamide and 0.1% *w*/*v* N-1-naphthylethylenediamine dihydrochloride) for 20 min at room temperature. The absorbance was measured at 550 nm using a SpectraMax M2 spectrophotometer (Molecular Devices, LLC., San Jose, CA, USA). The cell viability assay was performed using the Cell Counting Kit-8 (Dojindo Laboratories, Kumamoto, Japan). After treatment with the test samples and LPS, the culture medium was removed. The culture medium and Cell Counting Kit-8 were added to each well and incubated for 2 h at 37 °C in a humidified atmosphere of 5% CO_2_/95% air. The absorbance was measured at 450 nm using an Emax (Molecular Devices, LLC.). The percentages of NO production compared to the control (LPS) were calculated.

### 2.4. RNA Isolation and Real-Time RT-PCR Analysis

Total RNA was extracted using Isogen II, according to the manufacturer’s instructions. cDNA synthesis was performed using the FastGene Scriptase II cDNA Synthesis Kit. Real-time PCR was performed using Taq DNA Polymerase with Standard Taq Buffer (NEW ENGLAND BioLabs, Ipswich, MA, USA) and EvaGreen (Biotium, Inc., Fremont, CA, USA) with a Thermal Cycler Dice Real-Time System (Takara Bio Inc., Shiga, Japan). The following primers were used: mouse iNOS: 5′-TGGAGCCAGTTGTGGATTGTC-3′ (sense), 5′-GGTCGTAATGTCCAGGAAGTAG-3′ (antisense); mouse TNF-α: 5′-GCCTCTTCTCATTCCTGCTTG-3′ (sense), 5′-GGCCATTTGGGAACTTCTCA-3′ (antisense); mouse β-actin: 5′-CGGTTCCGATGCCCTGAGGCTCTT-3′ (sense), 5′-CGTCACACTTCATGATGGAATTGA-3′ (antisense). The obtained values were normalized to β-actin mRNA levels. The 2^−ΔΔCt^ method was used to calculate relative expression levels.

### 2.5. Statistical Analyses

The data were analyzed using an analysis of variance (ANOVA) followed by Dunnett’s test. Statistical significance was set at *p* < 0.05.

## 3. Results

### 3.1. Effects of M. citrifolia Seeds Extract on Nitric Oxide Production

**Figure 1:**
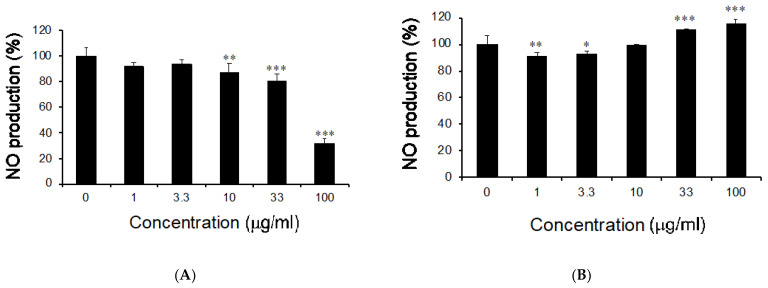
Inhibitory effects of *Morinda citrifolia* seeds extract or oil on NO production in LPS-stimulated RAW264 cells. Cells were treated with *M. citrifolia* (**A**) seed extract or (**B**) oil for 0.5 h before incubation with LPS (200 ng/mL). The indicated concentration shows the final concentration of the test samples incubated with LPS for 24 h. The level of NO in the culture medium was measured using the Griess reaction. Data are presented as mean ± standard deviation (SD) (*n* = 4). * *p* < 0.05, ** *p* < 0.01, *** *p* < 0.001 compared to the sample with 0 (zero) concentration.

We used RAW264 cells as an in vitro inflammatory model. Macrophages are activated by LPS release inflammatory mediators, such as NO and TNF-α, and the overproduction of NO is related to inflammation in abnormal situations [19]. Various natural extracts have been reported to exert anti-inflammatory effects. The LPS-stimulated RAW264 cells were treated with MCS-ext, MCS-oil, *M. citrifolia* leaf extract (MCL-ext), and *M. citrifolia* fruit extract (MCF-ext). The RAW264 cells were incubated with MCS-ext or MCS-oil for 0.5 h and treated with LPS in the presence of MCS-ext or -oil. The final concentration of MCS-ext or -oil was 0–100 μg/mL, and that of LPS was 200 ng/mL. The stimulation time of LPS was 24 h. NO production from LPS-stimulated RAW264 cells treated with MCS-ext or -oil was measured using the Griess reagent system. MCS-ext suppressed NO production in a dose-dependent manner (Figure 1A). MCS-oil slightly decreased NO production in low concentrations and increased the production in high concentrations (Figure 1B). The reason for this result is unclear, but MCS-oil had no dose-dependent effects on NO production.

**Figure 2:**
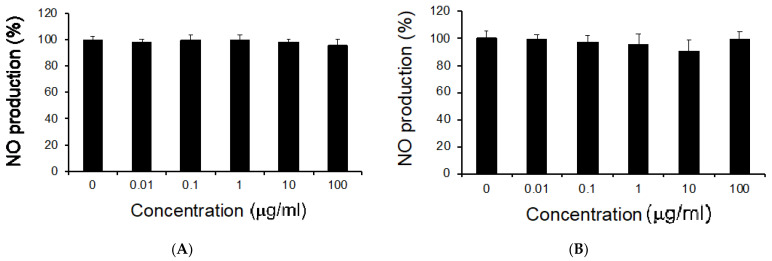
Inhibitory effects of *Morinda citrifolia* leaves or fruits extract on NO production in LPS-stimulated RAW264 cells. Cells were treated with the extract of *M. citrifolia* (**A**) leaves or (**B**) fruit for 0.5 h before incubation with LPS (200 ng/mL). The indicated concentration shows the final concentration of the test samples incubated with LPS for 24 h. The level of NO in the culture medium was measured using the Griess reaction. Data are presented as mean ± SD (*n* = 4).

Next, we examined whether MCL-ext or MCF-ext decreased NO production in LPS-stimulated RAW264 cells. We prepared freeze-dried juice from noni leaves and fruits to investigate the inhibitory effects of MCL-ext on NO production in LPS-simulated RAW264. MCL-ext and MCF-ext did not present inhibitory effects on NO production at concentrations up to 100 μg/mL (Figure 2). This result is almost consistent with previous reports showing the inhibitory effects of MCL-ext [15].

### 3.2. Effects of M. citrifolia Seeds Extract on Cell Viability

**Figure 3:**
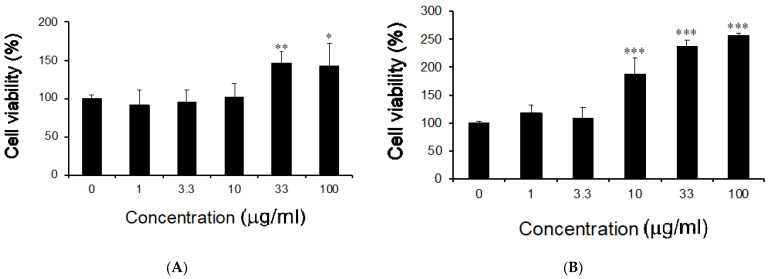
Effects of *Morinda citrifolia* seeds extract or oil on the cell viability in LPS-stimulated RAW264 cells. Cells were treated with *M. citrifolia* (**A**) seed extract or (**B**) oil for 0.5 h before incubation with LPS (200 ng/mL). The indicated concentration shows the final concentration of the test samples incubated with LPS for 24 h. Cell viability was measured using the Cell Counting Kit-8 assay. Data are presented as mean ± SD (*n* = 4). * *p* < 0.05, ** *p* < 0.01, *** *p* < 0.001 compared to the sample with 0 (zero) concentration.

We examined whether the MCS-ext treatment on LPS-stimulated RAW264 cells induced cytotoxic properties. RAW264 cells were incubated with the MCS-ext or MCS-oil for 0.5 h and treated with LPS in the presence of MCS-ext or -oil. The final concentration of MCS-ext or MCS-oil was 0–100 μg/mL, and that of LPS was 200 ng/mL. The stimulatory time of LPS was 24 h. To examine the cell growth of MC seeds, we used the Cell Counting Kit-8 assay to evaluate cell viability. Treatment with MCS-ext or -oil increased the viability of RAW264 cells stimulated with LPS as the concentration increased (Figure 3). When LPS-stimulated RAW264 cells were treated with 100 µg/mL MCS-ext, cell viability was improved by 50% as compared to the control. The results showed that the inhibitory effect of MCS-ext on NO production was observed in a range of no cytotoxicity.

### 3.3. Effects of M. citrifolia Seeds Extract on LPS-Induced Expression of Inflammatory Mediator

**Figure 4:**
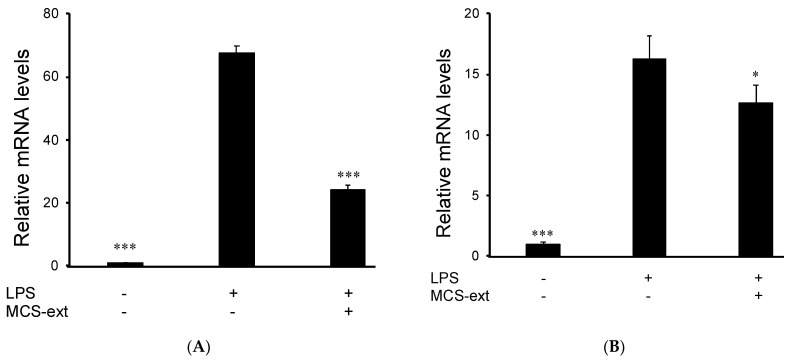
The effects of *Morinda citrifolia* seeds extract on iNOS and TNF-α expression in LPS-stimulated RAW264 cells. Cells were treated with MCS-ext (100 μg/mL) for 0.5 h before incubation with LPS (200 ng/mL). After 12 h of LPS stimulation, total RNA was isolated using Isogen II. (**A**) iNOS and (**B**) TNF-α mRNA levels were analyzed using real-time RT-PCR. The relative mRNA levels were determined using the *Ct* value method and normalized to β-actin expression. Data are presented as mean ± SD (*n* = 3). * *p* < 0.05, *** *p* < 0.001 compared to the LPS-treated group.

Additionally, we examined whether the treatment of MCS-ext in LPS-stimulated RAW264 cells modulated the expression of inflammatory factors. The mRNA levels of iNOS and TNF-α were measured using real-time RT-PCR. The transcription of iNOS is increased in activated macrophages [20], resulting in an increased NO production [21]. RAW264 cells were seeded and treated with 100 μg/mL MCS-ext for 0.5 h and treated with LPS in the presence of MCS-ext for 12 h. After stimulation, the cells were collected, and RNA was isolated. The LPS-induced expression of iNOS mRNA increased by 67.8 ± 1.91 times compared to that of nontreated control cells. However, the 100 μg/mL MCS-ext treatment before LPS stimulation decreased the expression level of iNOS to 24.3 ± 1.02 times that of the control cells (Figure 4A). Furthermore, we examined whether LPS-induced TNF-α expression could be regulated by treating LPS-stimulated RAW264 cells with MCS-ext. MCS-ext treatment decreased LPS-induced TNF-α upregulation in LPS-stimulated RAW264.

## 4. Discussion

We demonstrated that MCS-ext inhibited the LPS-induced inflammatory response in RAW264 cells. Previous reports have shown that noni juice from Costa Rica has anti-inflammatory and antioxidative effects and reduces carrageenan-induced paw edema [22] and lung inflammation sensitized by ovalbumin [23]. Noni leaf water extract improved the oxidative status and enhanced anti-inflammatory cytokine action [24]. MCL-ext has been reported to have anti-inflammatory effects in LPS-stimulated RAW264 cells in vitro [15].

Anti-inflammatory effects were determined by NO production and expression by the expression of TNF-α and iNOS. Our findings revealed that MCS-ext had the potential to suppress inflammation. TNF-α is a proinflammatory cytokine that contributes to extensive tissue damage; it is a mediator of the development of various inflammatory diseases [25] and plays a key role in the upregulation of other proinflammatory cytokines [26]. TNF-α is crucial for iNOS induction in LPS-stimulated macrophages [27]. We concluded that MCS-ext treatment reduced LPS-induced iNOS and TNF-α expression in activated RAW264 cells. These results suggest that MCS-ext reduces TNF-α expression and that the reduction of TNF-α suppresses iNOS expression in LPS-stimulated RAW264 cells. The detailed inhibitory mechanism of NO production by MCS-ext is unclear. It has been reported that compounds from Hawaiian noni fruit juice downregulated the expression of IKKα/β, I-κBα, and NF-κb p65 in LPS-stimulated macrophages [28]. Therefore, MCS-ext downregulating the expression of IKKα/β, I-κBα, and NF-κb p65 might inhibit the NF-κb signaling. In summary, MCS-ext exhibits anti-inflammatory effects at lower concentrations than that of MCF-ext, indicating that MCS-ext has a potential use as an anti-inflammatory agent for treating inflammation-related disorders.

## Data Availability

Not applicable.
